# Induction of Phage-Specific Antibodies by Two Therapeutic Staphylococcal Bacteriophages Administered *per os*

**DOI:** 10.3389/fimmu.2019.02607

**Published:** 2019-11-14

**Authors:** Joanna Majewska, Zuzanna Kaźmierczak, Karolina Lahutta, Dorota Lecion, Aleksander Szymczak, Paulina Miernikiewicz, Jarosław Drapała, Marek Harhala, Karolina Marek-Bukowiec, Natalia Jędruchniewicz, Barbara Owczarek, Andrzej Górski, Krystyna Dąbrowska

**Affiliations:** ^1^Hirszfeld Institute of Immunology and Experimental Therapy, Polish Academy of Sciences, Wrocław, Poland; ^2^Faculty of Computer Science and Management, Wrocław University of Science and Technology, Wrocław, Poland; ^3^Research and Development Center, Regional Specialist Hospital in Wrocław, Wrocław, Poland

**Keywords:** *Staphylococcus* phage, antibodies, immune response, oral administration, gastrointestinal tract, A3R phage, 676Z phage, translocation

## Abstract

In therapeutic phage applications oral administration is a common and well-accepted delivery route. Phages applied *per os* may elicit a specific humoral response, which may in turn affect phage activity. We present specific anti-phage antibody induction in mice receiving therapeutic staphylococcal bacteriophage A3R or 676Z in drinking water. The schedule comprised: (1) primary exposure to phages for 100 days, followed by (2) diet without phage for 120 days, and (3) secondary exposure to the same phage for 44 days. Both phages induced specific antibodies in blood (IgM, IgG, IgA), even though poor to ineffective translocation of the phages to blood was observed. IgM reached a maximum on day 22, IgG increased from day 22 until the end of the experiment. Specific IgA in the blood and in the gut were induced simultaneously within about 2 months; the IgA level gradually decreased when phage was removed from the diet. Importantly, phage-specific IgA was the limiting factor for phage activity in the gastrointestinal tract. Multicopy proteins (major capsid protein and tail morphogenetic protein H) contributed significantly to phage immunogenicity (IgG), while the baseplate protein gpORF096 did not induce a significant response. Microbiome composition assessment by next-generation sequencing (NGS) revealed that no important changes correlated with phage treatment.

## Introduction

With antibiotic resistance spreading at an alarming rate and only a few novel classes of antibiotics discovered in the last decades (1–3), the need for alternative therapeutics to control bacterial infections is critical. Among difficult bacterial infections, methicillin-resistant *Staphylococcus aureus* (MRSA) is one of the major threats (4–6). Although MRSA strains are not usually characterized by higher virulence than methicillin-sensitive strains (MSSA), they often carry concomitant resistance to many commonly used as well as new antibiotics and last-resort drugs (7–10), which greatly limits therapeutic options, and is associated with increased mortality risk and higher costs of treatment. Molecular characterization, evolution, and epidemiology of MRSA were recently thoroughly reviewed elsewhere (11).

Phage therapy is gaining renewed interest as alternative treatment in antibiotic-resistant bacterial infections (12–14). This includes phage therapeutics active against *Staphylococcus aureus*. Efficacy of staphylococcal phage therapy has been reported in cases of, among others, purulent disease of lungs and pleura (15), gastrointestinal infections, ulcerated varicose, pericarditis, and furunculosis (16, 17). Case studies have also demonstrated successful topical use of anti-staphylococcal phages in recurrent corneal abscess and interstitial keratitis caused by vancomycin-intermediate sensitivity *S. aureus* (VISA) (18), and orally as a three-phage cocktail (including phage A3R) in decolonization from MRSA in a healthcare worker of an intestinal carrier status (19). Staphylococcal phage ISP was proposed as a component of phage cocktail dedicated to treatment of infected burn wounds (20), but the preliminary, small-scale results were not satisfactory. In the Phage Therapy Unit of the Hirszfeld Institute of Immunology and Experimental Therapy (HIIET) phages specific to staphylococci have been applied most frequently (21–23). Both poly- and monoinfections with *S. aureus* were treated, including MRSA, and applied bacteriophages included A3R phage and 676Z phage (24). They have also been used as components of the therapeutic anti-staphylococcal phage cocktail MS-1 (25). Reports from the therapeutic use of these bacteriophages support good applicability of their oral use (21–23), which is also a convenient, relatively safe route of delivery, well-accepted by patients.

The role of phages in medicine is not restricted to their inherent ability to specifically infect and kill bacterial cells. Phages have recently been recognized as important components of the natural microbiome of humans and animals (26, 27), with a special focus on the gastrointestinal (GI) tract, where they constitute a major fraction of the virome (28, 29). Both natural bacteriophages and those introduced to humans or animals for therapeutic purposes enter interactions with mammalian immune systems. Although it is generally clear that bacteriophages are able to elicit specific antibody production which may in turn affect phage activity (30–39), the mechanisms and consequences of this process are vague. Factors that determine phage immunogenicity, and how specific responses affect phage pharmacokinetics, are not well-recognized either in exogenous (e.g., therapeutic) phages or those constituting a part of the natural virome (e.g., in the gut). In the first oral phage safety trial in humans (40) no anti-phage IgG, IgM, or IgA antibodies were detected in the blood of human volunteers receiving T4 phage for two consecutive days. Animal models, however, demonstrated that antibody induction by orally administered phages was possible by continuous exposure of animals to relatively high doses of phage (41). It has been documented that therapeutic phages induced phage-specific IgG, IgM, and IgA antibodies with development of phage-neutralizing activity in human blood (neutralization assessed *in vitro*); however, no correlation was observed between the induction of these phage-neutralizing antibodies and the outcome of phage therapy (25, 42). In early studies by Bradley et al. (43) actinophage MSP8 (*Siphoviridae*) was administered in drinking water to mice. Phage-neutralizing antibodies were detected in the blood, with the initial response involving 19s globulin (IgM), later accompanied by 7s globulin (IgG). Similar observations were reported for coliphage T4, which induced specific IgG after oral administration in mice (41). Notably, in these murine models phage doses were very high in comparison to those applied in humans. Interestingly, effective intragastric immunization does not require phage particles to be infective, as demonstrated by immunization with UV-inactivated filamentous phage (44); thus effective delivery of phage antigens can be sufficient for induction of the specific response.

The long-term kinetics of phage-specific antibody induction by orally applied model phage T4 so far have been demonstrated only in our previous studies (41). Continuous exposure of mice to relatively high phage titers in drinking water resulted in the induction of secretory anti-phage IgA in the gastrointestinal tract and anti-phage IgG antibodies in the blood. Phage-specific secretory IgA in the gut appeared to be the major factor limiting phage activity in the gut. Notably, secretory IgA decreased over time once the phage was removed from the diet. A significant increase of anti-phage IgM antibodies was not detected, and phage-specific IgA antibodies in the blood were not investigated. Differences in immunogenicity of capsid proteins were demonstrated in the model phage, where either high- or low-immunogenic proteins were identified. No other bacteriophages, including therapeutic ones, have been evaluated so far. Comparison of humoral response kinetics to phages representing different groups (e.g., naturally occurring vs. therapeutic ones) may allow to identify possible general trends in antibody induction by phages.

Here we present an immunological study of two related therapeutic bacteriophages active against *Staphylococcus aureus*: A3R and 676Z (23, 45) in a mouse model. A very long-term experiment (~9 months) allowed for studies of specific antibodies' complete kinetics: (1) in the prolonged primary exposure to phages, (2) during the time of phage removal from the diet, and (3) in the second exposure to the same phage. The study included both induction of specific secretory IgA and specific IgG, IgM, and IgA in blood, and further, it included bioavailability of active phage in relation to the kinetics of antibody production. Major phage structural proteins that contributed to specific antibody induction by phages A3R and 676Z were identified, and overall safety assessment was conducted with testing for microbiome composition in animal feces.

## Materials and Methods

### Bacteriophages and Bacterial Strains

A3R and 676Z phages were obtained from the Therapeutic Phage Collection of the Hirszfeld Institute of Immunology and Experimental Therapy (HIIET) PAS and propagated on *Staphylococcus aureus* strains R19930 and Z11778, respectively, both obtained from the Polish Collection of Microorganisms (HIIET PAS, Poland) and isolated from patients of the Phage Therapy Unit at the HIIET PAS.

Crude phage lysates were prepared as follows: flasks containing enriched nutrient broth were inoculated with bacterial host suspension and incubated at 37°C for 3 h with vigorous shaking. Then, phages were added to the flasks, and the cultures were kept at room temperature for 30 min to allow for phage adsorption and incubated at 37°C with vigorous shaking for 10 h. After that time the flasks were transferred to 4°C and left for 2 days to clarify. Phage lysates were then centrifuged at 8,000 rpm, and the supernatants were filtered through 0.22 μm Millipore membrane filters (Merck Millipore) and purified using size exclusion chromatography on Sepharose 4B (Sigma-Aldrich). Such preparations were either used to prepare phage-enriched drinking water for animal experiments, or subjected to dialysis through 1,000 kDa membranes (Spectrum Laboratories, USA) against PBS. For ELISA tests involving fecal samples, an additional purification step involving CIMmultus QA-1 Advanced Composite Column, pore size 2 μm (BIA Separations), was completed prior to dialysis. Phage titers in lysates and purified preparations were determined using serial dilutions and the double-layer agar plate technique according to Adams (46).

### Phage Proteins

Phage proteins were used as bottom antigens in ELISA immunoassay and to obtain protein-specific sera. Three proteins were used: Mcp—major capsid protein (product of gene AFN38122.1 or AFN38316.1 for A3R and 676Z, respectively); TmpH—tail morphogenetic protein H (product of gene AFN38181.1 or AFN38375.1 for A3R and 676Z, respectively); gpORF096 (product of gene AFN38152.1 or AFN38346.1 for A3R and 676Z, respectively). Genes were cloned in the Gateway technology into the expression vector (Thermo Fisher) pDEST15 (Mcp) or pDEST24 (TmpH and gpORF096) and expressed in *E. coli* B834(DE3) F^−^ *ompT* *hsdS*_*B*_(${{\text{r}}_{\text{B}}}^{-}$ ${{\text{m}}_{\text{B}}}^{-}$) *gal dcm met* (DE3) (Novagen) grown in Luria-Bertani Broth (LB) high salt (10 g/L of NaCl) culture medium (Sigma-Aldrich) supplemented with ampicillin, chloramphenicol and 3 mM L-arabinose (Sigma-Aldrich) at 37°C until OD_600_ reached 0.8. To facilitate proper folding of proteins, chaperones groES+groEL (from pGRO7 vector, TaKaRa Bio Inc.) were used. Expression of phage proteins was induced with 0.2 mM isopropylthio-b-D-galactoside (IPTG) (Thermo Scientific) and conducted overnight at 25°C. Cultures were then centrifuged for 5 min at 8,000 rpm and the supernatant was removed. Harvested bacteria were suspended in phosphate buffer (50 mM Na_2_HPO_4_, 300 mM NaCl, pH 8.0), treated with PMSF (1 mM) and incubated on ice for 15 min. The lysis was done by incubation with lysozyme (0.5 mg/ml) for 6–7 h on ice and by the freeze-thaw method (−80°C). The preparation was then supplemented with Mg^2+^ (up to 0.25 mM), DNase (10 μg/ml) and RNase (20 μg/ml), and incubated on ice for 3 h. Fractions were separated by two centrifugations (12 000 rpm, 45 min, 15°C). The soluble fraction was filtered through 0.45 μm PVDF filters and incubated with glutathione sorbent slurry (Glutathione Sepharose 4B, GE Healthcare Life Sciences), washed with phosphate buffer, and proteins were released by proteolysis with rTev protease (5 U/mL) (Pure Biologics, Poland) at 10°C; GST tags remained bound in the resin. LPS removal from all protein preparations was done with EndoTrap HD (Hyglos GmbH, Germany). Gel filtration FPLC (fast protein liquid chromatography) on a Superdex 75 10/300 GL column (GE Healthcare Life Sciences, Poland) was applied for final separation and proteins were dialyzed against PBS and filtered through 0.22 μm PVDF filters (Merck Millipore, Germany). Proteins were assessed by SDS-PAGE and concentrations were determined by the Lowry chromogenic method (Thermo Scientific, USA). Alternatively, protein concentrations were calculated based on SDS-PAGE protein band density with reference to a standard band of known protein concentration using dedicated software (GeneSnap, Syngene).

### Immunization of Mice

C57BL/6J normal male mice (*N* = 6 or 7) were obtained from the Medical University of Bialystok and bred in the Animal Breeding Centre of the Hirszfeld Institute of Immunology and Experimental Therapy (HIIET) in an isolated area in SPF conditions. All animal experiments were performed according to EU directive 2010/63/EU for animal experiments and were approved by the 1st Local Committee for Experiments with the Use of Laboratory Animals, Wrocław, Poland (project no. 76/2011). The authors followed the ARRIVE (Animal Research: Reporting of *in vivo* Experiments) guidelines.

Microbiological assessment confirmed that no phages active against the two host *S. aureus* strains were initially present in fecal and blood samples collected from the mice used for these experiments. Size-exclusion chromatography-purified preparations of phage A3R or 676Z in 0.068 M phosphate buffer (pH 7.2) were diluted in PBS and mixed with drinking water (1:1 ratio) to a final concentration of 4 × 10^9^ pfu/ml. Water mixed with PBS was used to maintain higher ionic strength since decreased ionic strength was shown to trigger phage aggregation (47). Mice were given such preparations continuously for 100 days as a sole water source. Phages were then removed from the diet and the experiment was continued for 120 days. After that, drinking water was once again replaced with phage preparations continuously for the next 44 days. Whenever phage-treated groups were given phage preparations, the control group was given drinking water mixed with PBS and phosphate buffer in the same ratio as phage-treated groups. Mice from phage-treated groups were separated from control mice.

Blood from the tail vein and fecal samples were collected throughout the experiments to determine the number of viable phage particles in feces and assess phage translocation to the circulation system. Active phages were detected in any samples when fresh only. Plasma was separated from blood samples by double centrifugation (2,250 g and 10,000 g) and stored at −20°C along with fecal samples for the subsequent evaluation of specific anti-phage antibody levels or microbiome assessment by 16S RNA analysis in next-generation sequencing (NGS).

### Intraperitoneal Immunization of Mice and Phage-Specific Reference Plasma Samples

C57BL/6J normal male mice (*N* = 5 or 6) (Medical University of Bialystok) were administered intraperitoneally (IP) three doses of highly purified phage preparations, 10^10^ pfu/mouse each on days 0, 20, and 50. Blood samples were collected from the tail vein on day 100 and plasma was separated as described above.

To obtain phage-specific reference plasma samples for the standard curve in calculation of ELISA units (41, 48, 49), C57BL/6J normal male mice (Medical University of Bialystok) were administered subcutaneously (s.c.) three doses of highly purified phage preparations, 5 × 10^10^ pfu/mouse each on days 0 (+ adjuvant), 20 (+ adjuvant), and 40. Blood was collected from the orbital vein on day 48.

### Protein-Specific Sera

Ovalbumin (OVA) was purchased from Abnova. Phage structural proteins Mcp, TmpH, and gpORF096 were obtained as described above. To obtain OVA-specific IgG, C57BL/6J male mice (Mossakowski Medical Research Centre, Polish Academy of Sciences, Warsaw) were administered subcutaneously (s.c.) three doses of OVA, 150 μg/mouse each on days 0 (+ adjuvant), 14, and 28. Blood was collected from the orbital vein to microtubes containing serum gel with clotting activator (Sarstedt, Germany) 8 days after the last dose. Serum was separated from blood by centrifugation (10 min, 2,000 g) and stored at −20°C for further use. To obtain phage protein-specific sera, C57BL/6J male mice were administered s.c. three doses of highly purified proteins Mcp, TmpH, or gpORF096, 200 μg/mouse each on days 0 (+ adjuvant), 21 (+ adjuvant), and 45. Blood was collected from the orbital vein 1 week after the last dose and serum was separated and stored as described above.

### Specific Anti-phage Antibody Level Measurement by ELISA

MaxiSorp flat-bottom 96-well plates (Nunc, Thermo Scientific) were coated with highly purified phage preparations 5 × 10^8^ pfu/well in 100 μl (or 7.5 × 10^8^ pfu/well in the case of fecal IgA assessment) overnight at 4°C. Subsequently, wells were washed 5 times with PBS or PBS with 0.05% Tween 20 (BD Biosciences) (in fecal samples) and blocked with 1% albumin (Sigma) in PBS at room temperature for 45 min. Blocking solution was then removed and plates were washed 5 times with PBS with 0.05% Tween 20. Plasma or fecal samples diluted in PBS were then added to wells at 100 μl per well and incubated at 37°C for 2 h. Samples were diluted as follows: 1:100, 1:200, and 1:400 for plasma IgM, IgG and IgA testing, respectively; 1:2,000 dilution was used for fecal IgA. Each sample was investigated in duplicate. Subsequently, plates were washed 5 (for plasma samples) or 7 (for fecal samples) times with PBS with 0.05% Tween 20 and 100 μl per well of diluted detection antibody: horseradish peroxidase-conjugated goat anti-mouse IgM (Jackson ImmunoResearch Laboratories), IgG (Jackson ImmunoResearch Laboratories), or IgA (BIO-RAD) was applied to the plates and incubated for 1 h at room temperature in the dark. The antibody solution was removed and the plates were washed 5 or 7 times with PBS with 0.05% Tween 20. TMB (50 μl/well) was used as a substrate reagent for peroxidase according to the manufacturer's instructions (R&D Systems) and incubated for 30 min. Finally, 25 μl of 2N H_2_SO_4_ was added to stop the reaction and the absorbance was measured at 450 nm (main reading) and normalized by subtracting the background absorbance at 570 nm.

To compare phage-specific IgG levels in plasma after different administration routes, a standard curve with phage-specific reference plasma samples was used as described previously (41, 48, 49). Each curve consisted of 10 points of 2-fold reference plasma dilutions, from 1:100 to 1:51,200 (each dilution was processed in duplicate) and two uncoated wells to which PBS was added instead of serum samples that served as blanks. Further steps of the assay were performed as described above. Gen5 was used to normalize and calculate ELISA units, with the standard curve as a reference (41, 48, 49). Plasma samples were diluted as follows: for 676Z phage 1:500 and 1:100 dilutions were used for phage-treated groups and control group, respectively, while for A3R 1:800 and 1:200 dilutions were used for phage-treated groups and control group, respectively. These dilutions were optimized to fit within standard curves in our previous immunological studies of phage-specific antibody production (data not shown). The dilution factor was taken into account when calculating final ELISA units.

### Assessment of Immunogenicity of Individual Proteins by ELISA

MaxiSorp flat-bottom 96-well plates (Nunc, Thermo Scientific) were coated with highly purified protein preparations. Each protein's individual ability to adhere to the plastic surface of the plate was assessed and normalized by the low-concentration CBQCA Protein Quantitation Kit (Thermo Fisher Scientific) (see below) and wells were covered overnight at 4°C with OVA (1.2 μg/well), Mcp (0.8 μg/well), TmpH (1.5 μg/well), and gpORF096 (0.2 μg/well). Subsequently, wells were washed 5 times with PBS and blocked with 5-fold diluted SuperBlock Blocking Buffer (Thermo Scientific) at room temperature for 45 min. Blocking solution was then removed and plates were washed 5 times with PBS with 0.05% Tween 20. Plasma samples diluted in PBS were then added to wells at 100 μl per well. Two standard curves were prepared for each plate: one with Mcp-, TmpH-, or gpORF096-coated wells and their respective reference sera, and one with OVA-coated wells and reference OVA-specific murine serum for normalization of results between plates. Each curve consisted of 10 points of 2-fold reference serum dilutions, from 1:100 to 1:51,200 (each dilution was processed in duplicate) and two uncoated wells to which PBS was added instead of serum samples that served as blanks. Further steps of the assay were performed as described above. Gen5 was used to normalize and calculate ELISA units, with the OVA standard curve as a reference (41, 48, 49).

### Blocking of Phage Activity by Murine Plasma Samples

Plasma samples collected from mice immunized with A3R or 676Z phages either *per os* or IP and from control mice were diluted 2,000-fold in PBS and incubated with equal volumes of purified phage preparations of phages A3R or 676Z (2 × 10^6^ or 1 × 10^7^ pfu/ml) for 30 min at 37°C. Then, samples were serially diluted and phage titers were determined using the spot plating technique. The plasma dilution factor used in this experiment was determined experimentally, as for dilutions smaller than 1:2,000 the difference between groups was either undetectable or unclear (for exemplary results see Supplementary Figure 7).

### Phage Translocation From GI Tract to Circulation

C57BL/6J normal male mice (*N* = 5, 6, or 7) (Mossakowski Medical Research Centre, Polish Academy of Sciences, Warsaw) were used. Size-exclusion chromatography-purified preparations of phage A3R or 676Z in 0.068 M phosphate buffer (pH 7.2) were diluted in PBS and mixed with drinking water to a final concentration of 4 × 10^9^ pfu/ml (“low”) or 8 × 10^10^ pfu/ml (“high”). Mice received these preparations as a sole water source for 30 h and food was removed for the initial 5 h. To assess the effect of gastric juice neutralization on phage translocation to blood, one of two groups receiving a high phage titer was given 200 mM sodium bicarbonate in drinking water for 16 h prior to the experiment. Blood samples were collected from the tail vein after 5 and 27 h of phage administration in drinking water. Phage titers in blood were determined using double-layer agar plates technique according to Adams (46).

### Measurement of Protein Content in Wells

The CBQCA Protein Quantitation Kit (Thermo Fisher) was adapted to measure protein content in wells of MaxiSorp flat-bottom 96-well plates and determine optimal protein concentrations for plate coating. Plates were coated with OVA and phage proteins at various concentrations overnight at 4°C. PBS was added to wells serving as blanks (6 wells) and designated for the standard curve. Plates were washed 5 times with PBS. Eighty microliters of assay buffer (0.1 M sodium borate buffer, 0.1% Triton X-100, pH 9.3) supplemented with 0.83 mM KCl were added to protein-coated and blank wells. A six-point standard curve of 160, 80, 40, 20, 10, and 5 ng per well of bovine serum albumin (BSA) in the assay buffer supplemented with 0.83 mM KCl was prepared in designated wells. Subsequently, 20 μl of 1 mM ATTO-TAG CBQCA reagent solution in the assay buffer were added to each well. The plate was shielded from light and incubated for 2 h at room temperature with shaking (400 rpm). Fluorescence was read using the excitation/emission wavelength of ~465/550 nm. The mean blank value was calculated and subtracted from the results. Protein content in OVA- and phage protein-coated wells was calculated with reference to the standard curve obtained for BSA.

### Microbiome Assessment of Mice

DNA isolation from murine feces was performed with the QIAamp DNA Stool Mini Kit (QIAGEN) preceded by physical homogenization with 0.1 mm zirconia beads (OPS Diagnostics) in 1 ml of InhibitEX buffer: samples were vortexed for 1 min, incubated in 95°C for 5 min, vortexed vigorously for 3 min and finally centrifuged for 1 min at 14,500 rpm. Further steps were performed according to the manufacturer's instructions followed by an additional cleaning procedure using Genomic DNA Clean & Concentrator-10 (Zymo Research). DNA was eluted with 200 μl of ATE buffer and samples were stored at −20°C for further use.

Further steps were performed as described previously (50). Briefly, preliminary measurement of DNA concentration in the samples was performed with the Qubit 2.0 fluorometer using the Qubit dsDNA HS assay kit. Three samples of the highest quality from each group were selected for further processing. The Ion 16S Metagenomics Kit (Thermo Fisher) was used to amplify DNA coding for 16S rRNA V2, V3, V4, V5, V6–7, V8, V9 hypervariable regions' coding sequences, according to the manufacturer's instructions, using 5 ng of DNA for each sample. Barcoded libraries were created by the Ion Xpress Plus Fragment Library Kit with Ion Xpress Barcodes. The final library concentration was quantified by RT-qPCR with the Ion Library TaqMan Quantitation Kit according to the manufacturer's protocol. Emulsion PCR and the bead enrichment step were performed on the Ion OneTouch 2 System with the Ion PGM Hi-Q View OT2 Kit. Enriched template beads were mixed with the reagents from the Hi-Q View 400 Sequencing kit and loaded onto Ion Torrent 314 V2 chips. Sequencing parameters standard for 16S rRNA Targeted Sequencing were used based on the manufacturer's protocol.

Unaligned binary data files [Binary Alignment Map (BAM)] generated by the Ion Torrent PGM were uploaded to IonReporter version 5.6. Analysis was performed with the base pair cut-off number set at 150, minimum alignment coverage at 90%, and minimum abundance at 10 copies. Curated MicroSEQ 16S Reference Library v2013.1 was used as a reference database to identify obtained reads. Results obtained by the described workflow were visualized by KRONA software integrated in IonReporter 5.6.

## Results

### Phage Passage Through the Gastrointestinal Tract and Induction of Phage-Specific IgA in Murine Gut

Before the experiment, microbiological assessment of bacterial colonies isolated from murine feces indicated lack of *S. aureus*, confirming that phages A3R and 676Z had no natural host within the GI tract of mice used in this experiment (data not shown). By the use of the animals with a natural microbiome (including virome) but without phage hosts, this study allowed for assessment of the phage-specific immune response and its impact on phage viability in the normal GI tract, without interfering effects of phage propagation on a sensitive host. The experiment was 264 days long and it comprised three stages: (I) on days 0–100 mice were continuously given purified A3R or 676Z phage preparations as a drinking solution at a final concentration of 4 × 10^9^ pfu/ml (which corresponds to ~2 × 10^10^ pfu per mouse daily as calculated from the average daily water intake); (II) on days 101–220 phages were removed from the diet, and then (III) phage was given again on days 221–264 the same way as at stage I.

**Figure 1:**
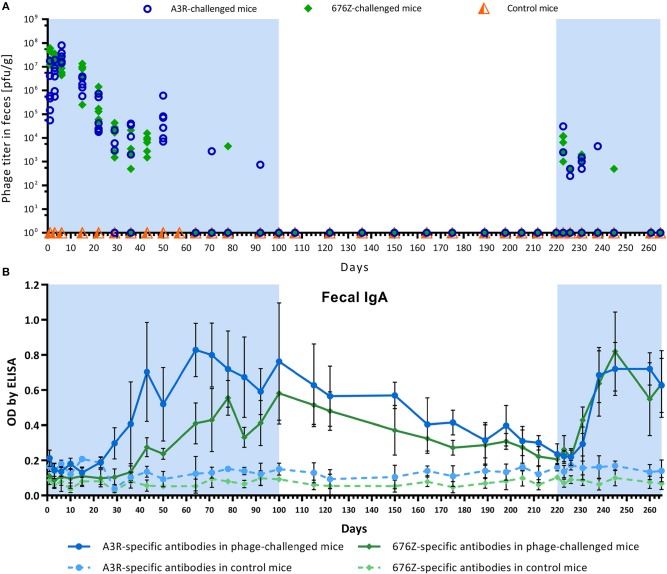
Phage transit through the GI tract **(A)** and changes of specific anti-phage IgA levels in murine fecal samples **(B)** in mice treated with phages A3R or 676Z. Antibody levels were evaluated using enzyme-linked immunosorbent assay (ELISA). Mice (*N* = 6) were administered purified preparations of phages A3R or 676Z in drinking solution of the final concentration 4 × 10^9^ pfu/ml. Phage-enriched drinking water was administered as the only water source continuously for 100 days. For the following 120 days phage preparations were removed from the diet and then introduced again for the final 44 days of the experiment. The periods of phage treatment are indicated in the figure as a light blue background. Control mice were separated from phage-treated mice and received no phage in the diet. They were examined for presence of phages active against A3R and 676Z bacterial *S. aureus* host strains and no phage activity was detected during the whole experiment. The experiment was repeated twice with concordant results. One representative experiment is presented in the figure.

Passage of active phages through the GI tract was assessed by the phage recovery from feces; their kinetics are presented in Figure 1A. Primary exposure (stage I) to phages resulted in very effective gut transit. Phage titers recovered from murine feces within the first 24 h reached 3.84 × 10^6^ and 3.53 × 10^7^ for A3R and 676Z, respectively. Within the next 2 weeks of phage treatment, phage titers recovered from feces varied in the range 1.9 × 10^6^ to 2.5 × 10^7^ pfu/g and 7.5 × 10^6^ to 3.5 × 10^7^ pfu/g for A3R and 676Z, respectively. This correlated with very low secretory IgA levels (Figure 1B; the same timescale was used as for phage recovery in Figure 1A). Later, IgA was gradually increasing, and that correlated with gradually decreasing phage titers in feces. Phages were still detectable until day 50, ranging between ~5 × 10^3^ and 5 × 10^5^ pfu/g, which is 2–4 orders of magnitude less than at the beginning of the experiment. From day 64, phages were undetectable or single plaques in single individuals were observed. This correlated with markedly increased levels of phage-specific secretory IgA that were observed approximately from 2 months of primary phage treatment until its end (day 100) (Figure 1).

During the second stage of the experiment (days 101–220), when phage was removed from the diet, phages were not detected in feces (as expected, also with regard to the lack of a sensitive bacterial host). Importantly, when animals were not exposed to the phages, phage-specific IgA in feces gradually decreased to insignificant levels within the next 120 days (on day 220) (Figure 1). During the third stage of the experiment, when phage preparations were again added to drinking water, phage-specific secretory IgA increased sooner, i.e., within the next 2 weeks, but before they did, active phages were able to pass the GI tract again. Nevertheless, mean titers were markedly lower, in the range 1.3 × 10^2^ to 6 × 10^3^ and 8.3 × 10^1^ to 5.6 × 10^3^ pfu/g for A3R and 676Z, respectively, and they decreased to undetectable levels within only 25–30 days. Again, significantly elevated secretion of phage-specific IgA correlated with the lack of active phage shedding.

### Induction of Anti-phage Antibodies in the Blood of Mice Treated With Phage Preparations *per os*

Administration of the phages in drinking water to mice allows for detection of active phage in blood, but detection is irregular (not in all individuals) and in low concentrations. Surprisingly, even administration of phages at the concentration of 8 × 10^10^ pfu/ml often did not result in detectable levels, and the maximum blood titer achieved in some individuals did not exceed 2 × 10^3^ pfu/ml (Supplementary Figures 1, 2). Importantly, any phages were detectable in blood only within the 1st week of the treatment (Supplementary Figure 2), which may be related to gradually increasing specific antibody levels in blood.

**Figure 2:**
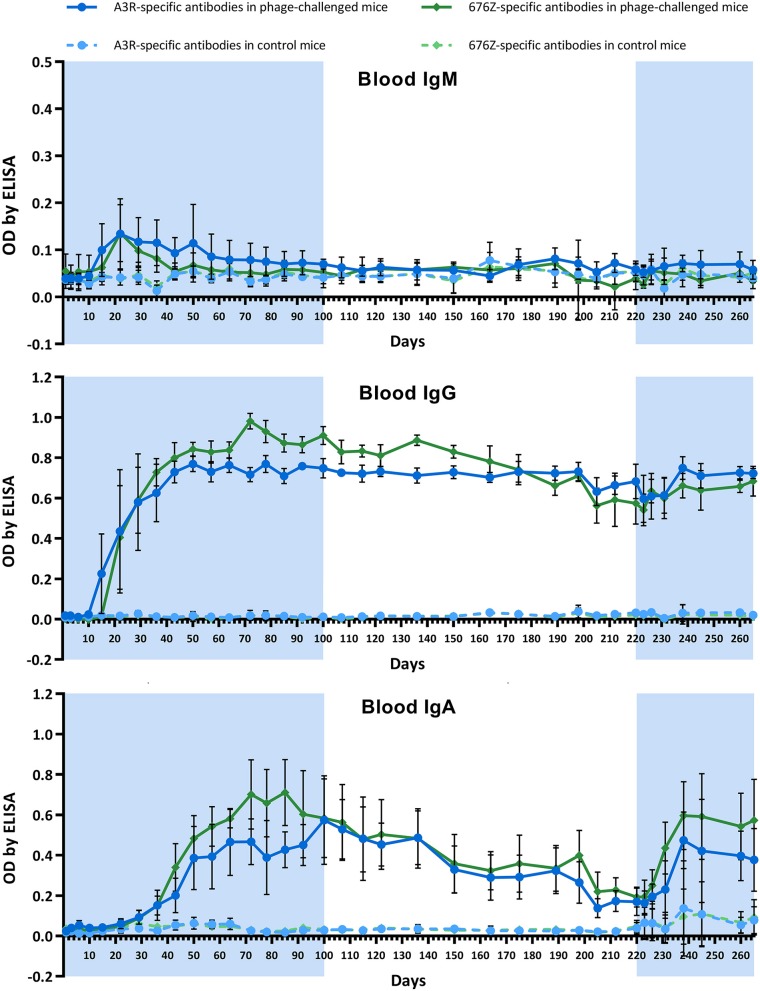
Kinetics of phage-specific antibody production in blood of mice treated with phages A3R or 676Z. IgM, IgG, and IgA levels were evaluated by enzyme-linked immunosorbent assay (ELISA). Mice (*N* = 6) were administered purified preparations of phages A3R or 676Z in drinking solution of the final concentration 4 × 10^9^ pfu/ml. Phage-enriched drinking water was administered as a sole water source continuously for 100 days. For the following 120 days phage preparations were removed from the diet and then applied again for the final 44 days of the experiment. The periods of phage treatment are indicated in the figure as a light blue background. Control mice were separated from phage-treated mice and received no phage in the diet. They were examined for presence of phages active against A3R and 676Z bacterial *S. aureus* host strains and no phage activity was detected during the whole experiment. Blood was collected from the tail vein; thus the same mice were sampled for the whole experiment. The experiment was repeated twice with concordant results. One representative experiment is presented in the figure.

During the whole experiment, animals were repeatedly tested for A3R and 676Z phage-specific antibodies in blood (plasma). Kinetics of IgM, IgG, and IgA production are shown in Figure 2. In general, a clear increase of phage-specific antibody levels in all classes was observed. No significant differences between the two investigated phages were observed. The pattern of antibody increase was typical for many proteinaceous antigens capable of induction of a T-dependent antibody response. First, phage-specific IgM increased from ~day 10, reaching a maximum concentration in plasma on day 22. After that, phage-specific IgM gradually decreased to insignificant levels. In parallel to the increase of IgM, phage-specific IgG also began its increase; it reached a significantly elevated concentration on day 22 and continued its intensive increase for approximately a month (until day 50), when it achieved a roughly stable, markedly high level. This high concentration did not decrease significantly until the end of the experiment, even at stage II when phage preparations were not administered to the animals (Figure 2). Concordantly, second administration of the phages on day 220 (stage III of the study) did not result in detection of active phage particles in blood (data not shown). At the same time, phage passage through the gut was effective again (Figure 1A).

Phage-specific IgA levels in blood (Figure 2) were consistent with those observed as secretory antibodies in feces (Figure 1B): phage-specific IgA levels were not detectable in blood at the beginning of the experiment, then gradually increased, reaching their highest levels after ~2 months of treatment. After phage removal from the diet, IgA levels gradually decreased, reaching a markedly lower concentration on day 220 (day 120 of the pause in phage treatment). Notably, in contrast to IgA, IgG level was not affected by removing phage from the diet. The second administration of phage preparations induced anti-phage IgA production much sooner than primary administration: it reached significantly increased levels within the next 2 weeks (Figure 2), which was concordant with secretory IgA induction (Figure 1B).

### Individual Immunogenicity of Selected Structural Proteins: Major Capsid Protein (Mcp), Tail Morphogenetic Protein H (TmpH), and Baseplate Protein gpORF096

Structural phage proteins may differ significantly in their ability to induce a specific humoral response, as demonstrated previously in T4 phage (37, 41). Therefore, immunogenicity of two structural proteins abundantly present on the A3R and 676Z phage virions was investigated: Mcp—major capsid protein (product of gene AFN38122.1 or AFN38316.1 for A3R and 676Z, respectively); and TmpH—tail morphogenetic protein H (product of gene AFN38181.1 or AFN38375.1 for A3R and 676Z, respectively) (45). In addition, gpORF096 (product of gene AFN38152.1 or AFN38346.1 for A3R and 676Z, respectively) was investigated as a recently identified element of phage baseplate necessary for effective infection of host bacteria (51). All three proteins share 100% amino acid sequence identity between A3R and 676Z phages.

**Figure 3:**
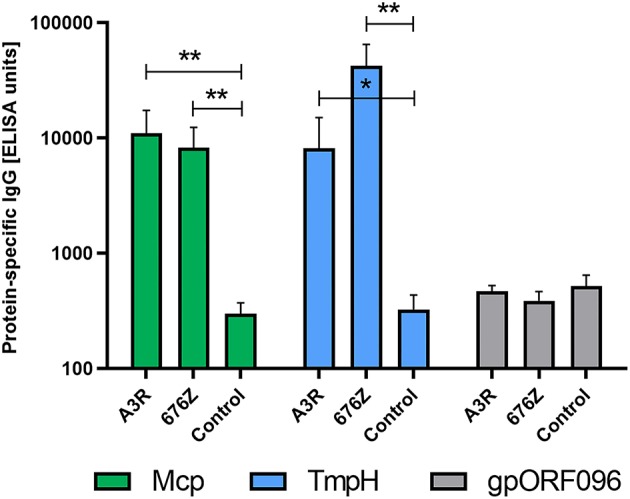
Individual immunogenicity of three selected structural proteins of phages A3R and 676Z in mice treated with the phage *per os* assessed by IgG ELISA units. Mice (*N* = 6) were administered purified preparations of phages A3R or 676Z in drinking water mixed with PBS to a final concentration of 4 × 10^9^ pfu/ml. Phage-enriched drinking water was administered as a sole water source continuously for 100 days. Separated plasma samples from these mice were examined for IgG antibodies specific to selected structural proteins: Mcp, TmpH, and gpORF096. Results were normalized and ELISA units were calculated with regard to a standard curve obtained for OVA-coated wells and OVA-specific murine serum (41, 48, 49). Statistically significant differences between groups are marked with asterisks: *p < 0.008, **p < 0.005 (one-tailed Mann-Whitney *U*-test).

IgG antibodies specific to the structural proteins Mcp, TmpH and gpORF096 were analyzed on day 100, when the specific response to phages was fully developed. They were analyzed by ELISA, and to allow for comparisons, results were normalized as ELISA units (48, 49). A marked increase of both Mcp- and TmpH-specific antibodies was observed, both in A3R and in 676Z phage-treated mice (in comparison to control non-treated mice) (Figure 3), which demonstrates that major capsid protein and tail proteins importantly contribute to the overall immunogenic effect of these phages. This is in line with the high copy number of these two proteins on bacteriophage virions. By contrast, no increase in protein-specific antibodies was observed for the less abundantly present baseplate protein gpORF096 (Figure 3).

### Comparison of Phage-Specific IgG Induction in Phage Treatment by Different Administration Routes

**Figure 4:**
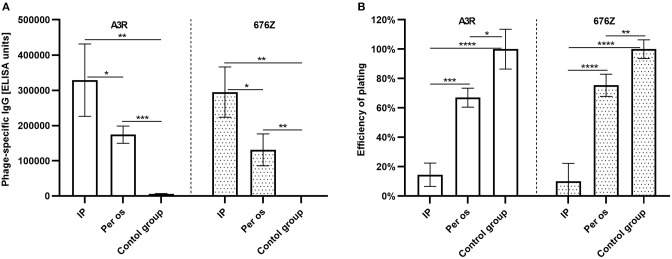
Comparison of A3R and 676Z phage immunogenicity in different administration routes: intraperitoneal (IP) and *per os*. Intensity of anti-phage IgG induction by A3R and 676Z phages applied *per os* or intraperitoneally **(A)**, and efficiency of plating of A3R and 676Z phages with plasma samples from mice treated with A3R or 676Z phage *per os* and intraperitoneally **(B)**. Mice (*N* = 5, 6, or 7) were administered purified preparations of phages A3R or 676Z in drinking water as a sole water source continuously for 100 days or they were injected with the phages intraperitoneally (IP). For oral treatment a dose of 4 × 10^9^ pfu/ml was used, thus making ~2 × 10^10^ pfu/mouse daily, 2 × 10^12^ in total. Intraperitoneal immunization was done with three successive injections of 1 × 10^10^ pfu/mouse on days 0, 20, and 50. Antibody levels were tested in samples collected on day 100. Results were normalized and ELISA units were calculated with regard to a standard curve obtained for reference phage-specific sera (41, 48, 49). EOP was tested for plasma samples collected on day 100 and diluted 2,000-fold. Exemplary results are presented. Statistically significant differences between groups are marked with asterisks: *p < 0.04, **p < 0.006, ***p ≤ 0.0003, ****p < 0.0001 (one-way ANOVA Brown-Forsythe and Welch test; outliers were excluded).

Effect of administration route on A3R and 676Z phage ability to induce a specific response was assessed. Anti-phage IgG levels in mice treated with A3R or 676Z phage *per os* (4 × 10^9^ pfu/ml, which corresponds to ~2 × 10^10^ pfu/mouse daily, continuously for 100 days) were compared to those of mice injected with the phage intraperitoneally (three doses, 1 × 10^10^ pfu/mouse each). Injection resulted in 1.9 and 2.2 times higher immunization than the oral route for A3R and 676Z phage, respectively (Figure 4A). This correlated with differences in phage-neutralizing activity of plasma, as determined by efficiency of plating (EOP): EOP for A3R and 676Z phages incubated with IP-developed specific plasma samples was only 15 and 10% (respectively), while it was 67 and 75% after incubation with samples from orally immunized mice (Figure 4B). Thus, neutralization was significantly stronger when animals were treated with phage IP than in those treated orally, even though the total dose of phage was almost two orders of magnitude higher in the latter ones.

## Discussion

In this study we investigated two similar staphylococcal bacteriophages, A3R and 676Z, with high therapeutic applicability (23, 24, 45). We compared how these phages induce a specific immune response (antibodies) when administered orally, and how in turn the specific immune response affects phage bioavailability in the GI tract. We observed that both bacteriophages induced specific antibody production in the gut (secretory IgA) and in the blood (IgG, IgM, IgA) (Figures 1, 2, respectively). Induction of secretory IgA antibodies was rather slow, and it took more than a month of intensive exposure to phage (4 × 10^9^ pfu/ml in drinking water, continuously) to achieve a notable increase in fecal phage-specific IgA. A substantial increase was observed after 2 months of exposure to the phages. This suggests that phages, although immunogenic, were not very effective in the local (mucosal) induction of a specific immune response when given orally. However, once A3R- and 676Z-specific IgA antibodies were efficiently produced, gut passage of active bacteriophages was inhibited (Figure 1). Importantly, when bacteriophages were removed from the diet, secretory IgA levels in the gut gradually decreased to insignificant levels, which again allowed for the passage of active phages through the gut. This second introduction of phage to the diet induced specific IgA sooner, but still it took ~2 weeks. For that duration, active phages were again detected in fecal samples (Figure 1). Thus, specific IgA secreted in the gastrointestinal tract was the limiting factor for phage viability there, and for effective gut passage. Gut passage was independent of phage-specific IgG production in blood, which was also observed. IgG was significantly increased from about day 22, and it remained at a high level until the very end of the experiment (Figure 2).

We demonstrated that phage-specific IgG rich plasma samples from mice immunized orally had phage neutralizing activity *in vitro*. The presence of specific antibodies in the circulation has been shown to affect phage pharmacokinetics and result in neutralization of phage particles by specific sera as well as in decrease of the efficacy of phages against bacterial pathogens in animal models (39, 52–54). Here we also observed that once the level of phage-specific secretory IgA in the gut increased significantly, bioavailability of orally administered phages in the GI tract decreased dramatically as no active phage particles were detected in feces (Figure 1). Thus, our animal model demonstrates that specific immune response is capable of decreasing bactericidal activity of bacteriophages *in vivo*.

These observations are concordant with previous observations of specific antibody induction (in the same model) by a model phage: coliphage T4 (41). T4, when applied in the same dose, induced specific IgA in the gut in a very similar time (more than a month). When the phage was removed from the diet, T4-specific IgA decreased to insignificant levels within about 100 days, which allowed for efficient gut passage of active phages through the gut. Further, second exposure to the phage induced phage-specific IgA much sooner than during the first exposure to phage (within about 2 weeks) and later active T4 phage was no longer detected in fecal samples. Gut passage of active T4 was also independent of phage-specific IgG in blood; IgG was significantly increased from day 36, and it remained at a high level until the very end of the experiment (41). Coliphage T4 was used in the previous study as a model phage. However, although T4-like phages are considered as therapeutic candidates, T4 itself has never been adapted for phage therapy, partially because of the high prevalence of T4-specific antibodies in the human population (37). Since observations for the coliphage T4 and for taxonomically irrelevant phages A3R and 676Z are so similar, the observed schema of immune responses to these phages seems to be (at least to some extent) universal and can also apply to other phages. At least phages of overall similar structure and chemistry (DNA, tiled) may share similar overall immunogenicity and resulting kinetics of immune response and phage bioavailability. Of note, other administration schedules may result in different kinetics and we are going to investigate this issue in future studies.

In the case of phage-specific IgM, its increase was noted in this study (Figure 2), while in previous studies on T4 it was not detected (41). Phages A3R and 676Z induced specific IgM in blood at ~2 weeks of the treatment, the maximum being observed on day 22. The achieved peak was not very high (Figure 2), but observed phage-specific IgM and IgG levels represented the classical pattern of specific antibody induction by T-dependent antigens, where T cells provide essential costimulatory signals for B cell differentiation. In that pattern, B cells primarily generate specific IgM, but as the response progresses B cells undergo Ig class switching together with affinity maturation. They become long-living plasma and memory cells, and antibody production switches to high-affinity IgG and achieves a relatively high and stable level (55–58). The T-dependent immune response is typically expected for proteinaceous antigens; since proteins are major components of phage virions, phages comply with characteristics of T-dependent antigens. We suppose that also in previous experiment with T4 (41), even if not detected, T4-phage specific IgM were likely produced at a low level. One should expect IgM production also in response to other bacteriophage strains.

Exposure of a system to antigens delivered to the GI tract may result in the development of oral tolerance. This may be especially relevant in case of phages, as they are naturally present in the gut as a dominant fraction of the virome and hence may be subjected to mucosally induced tolerance. However, in our model of oral immunization we observed typical induction of phage-specific IgG antibodies on a systemic level within 2 weeks, followed by systemic and secretory phage-specific IgA later on. Therefore, we hypothesize that high dose of phages administered in the presented model was sufficient to prevent the development of tolerance to these phages. This observation is supported by the results of previous studies involving a model coliphage T4, as the induction of humoral response was shown to be dose-dependent: no secretory IgA in the gut and only slight increase in phage-specific IgG in the blood was detected when a 10-fold lower dose of phage was administered (41). Further, Flanagan et al. (59) highlighted differences between digested (fragmented) antigens that tend to remain tolerogenic, in the contrary to intact antigens that become immunogenic. Phages investigated herein were relatively resistant to digestion since they traveled through the GI tract remaining active, thus they are not fragmented. This may suggest that capability of phages to escape degradation in the GI tract predisposes them, on the other hand, to be immunogenic and to induce specific IgG and IgA (59).

At the structural protein level, major capsid protein and tail sheath protein importantly contributed to the overall immunogenic effect of both investigated bacteriophages. This is in line with the high copy number and strong exposure of these two proteins on bacteriophage virions. However, this observation is different to that in T4 phage, where the multicopy major capsid protein gp23 had much less effect on the humoral response in oral administration (41), although gp23 had been shown to effectively induce specific antibodies in IP treatment (37). An increase in the level of antibodies specific to the less abundant structural component baseplate protein gpORF096 was not observed, which suggests that this protein does not contribute to the overall phage immunogenicity in this model. This observation is important from the practical point of view, since anti-gpORF096 antibodies were shown to neutralize phage ability to infect sensitive bacteria, while Mcp- and TmpH-specific antibodies did not affect phage infectivity (51). It is possible that gpORF096 is involved in host recognition and phage binding to host receptors (51); therefore its low immunogenicity seems beneficial for the phage as it would aid in retaining the ability to infect its bacterial host and propagate.

Duration of exposure to phages is among major factors relevant for the therapeutic use of phages. The length of oral phage therapy varies, as both short and prolonged administration of phages has been described, up to months (23). For this reason, we aimed to develop a model that would comprise the assessment of prolonged oral treatment and resulting kinetics of phage-specific antibody production. Aiming to identify possible general trends in antibody induction by phages, the schedule used in this work was almost identical to that applied in our previous work with coliphage T4 (41) to allow for a comparison between phages representing different groups.

Possibly absorption of phage proteins is sufficient to induce phage-specific antibodies in blood. We find it unclear whether active phage translocation from gut to blood is necessary for development of a specific antibody response in the blood. Here we observed very poor and highly irregular translocation of active bacteriophages in both investigated phage strains (Supplementary Figures 1, 2). Based on *in vitro* cell culture assay, Nguyen (60) hypothesized that phage transcytosis is a common phenomenon and as many as 31 billion phage transcytotic events occur daily within the human body. This was calculated from the *in vitro* rate of phage transcytosis 0.325 × 10^−12^ ml/(μm^2^ × h), which in mice with a much smaller gut surface (61) and with average phage concentration in the intestine achieving 10^7^-10^8^ pfu/ml (as observed herein) may translate to more than one million transcytotic events daily (10^6^ pfu/day). Unexpectedly, only poor and irregular detection of active phages in blood occurred (Supplementary Figures 1, 2). Most individuals' blood samples contained no active phage; the highest phage titers in rare animals reached 10^2^-10^3^ pfu/ml (median in all experiments: 0, mean: 5 × 10^1^ pfu/ml) (Supplementary Figures 1, 2). We developed a model that accommodated rate of phage transcytosis as proposed by Nguyen et al. (60) together with experimentally identified kinetics of phage clearance from blood as reported by Kim et al. (62). This model was used to estimate expected phage concentrations in murine blood (for details see Supporting Material: *Mathematical model* and *A3R and 676Z phage translocation from GI tract to circulation*). Simulated (expected) phage amounts were approximately one order of magnitude higher than the experimentally observed ones (Supplementary Figure 5). This discrepancy can be partially explained by the fact that *in vitro* cell cultures provide very different conditions to gut in a living animal or human and transcytosis observed *in vitro* may differ from that *in vivo*. Furthermore, the digestive tract content being an absorbing matrix, and immune-related factors (including non-specific ones, such as phagocytes) may strongly decrease the amount of phages that are able to translocate to the circulation effectively. Probably, translocated bacteriophages are filtered by intestinal lymph nodes (63), which are able to prevent the spread of translocating virions throughout the whole body. As a result, active phage concentrations in blood achieved by oral application are much lower than expected. Importantly, in spite of poor translocation of active phage, phages effectively induced specific IgM, IgG, and IgA antibodies in blood. This suggests that systemic circulation of antigen-presenting cells and other immunological cells engaged in development of a specific immune response allows for full development of the antibody repertoire in the circulation.

The normal immune response and lack of visible side effects in phage-treated mice are evidence supporting the safety of the phage preparations used in this study. Normal animals with natural bacterial flora in the gut were microbiologically assessed and confirmed as lacking A3R- or 676Z-sensitive bacteria in the GI tract. Such a model allows for a direct assessment of immune responses to phage in normal animals, since they are colonized with a natural microbiome, which is known to be a key factor for proper development of immune responses. Furthermore, this model allowed for observation of the potential impact that the specific immune response exerted on phage viability in the GI tract, without interfering effects of phage propagation on sensitive hosts. For this reason, selection for phage-resistant bacteria in the gut microbiome was not possible and was not a subject of investigation. However, the general composition of fecal microbiome was assessed by 16S rRNA targeted sequencing and compared between day 1 and 100; it revealed no important changes correlated with phage treatment in the microbiome composition (Supplementary Table 1, Supplementary Figure 6). This further supports the safety of oral phage applications.

This study revealed that oral administration of bacteriophages induced only a weak response to the phage. In the case of secretory IgA, which was revealed as the limiting factor for phage activity in the gut, termination of exposure to phage results in a decrease of specific IgA. This restores the possibility for phage to pass actively through the gut. Together with the positive assessment of safety, this is encouraging for the perspective of therapeutic use of phages, especially in treatment of infections in the alimentary tract.

## Conclusions

The investigated bacteriophages A3R and 676Z when applied orally are capable but not very effective in induction of a specific immune response.

Specific anti-phage IgA in the gut is a major factor limiting the passage of active A3R and 676Z phages through the mammalian GI tract. Importantly, IgA levels may decrease to insignificant levels when exposure to phage is terminated. These effects seem to be universal, since the same effects were demonstrated previously for T4 phage. At least phages of overall similar structure and chemistry (DNA, tiled) may share similar overall immunogenicity and resulting phage kinetics.

Although phage translocation from gut to blood was poor and highly irregular, phage-specific IgM, IgG, and IgA were induced in blood.

Major capsid protein and tail morphogenetic protein H strongly contributed to the immunogenic effect of phages A3R and 676Z, while less abundantly represented baseplate protein gpORF096 was not immunogenic in this model.

## Data Availability Statement

The datasets generated for this study are available on request to the corresponding author.

## Ethics Statement

This study was carried out in accordance with the recommendations of EU directive 2010/63/EU. The protocol was approved by the 1st Local Committee for Experiments with the Use of Laboratory Animals, Wrocław, Poland.

## Author Contributions

KD and JM conceived and designed the experiments. ZK cloned phage genes and optimized expression and purification of phage protein preparations. JM, KD, KL, DL, AS, PM, MH, KM-B, NJ, and BO performed the experiments. KD and JM analyzed the data. JD provided the mathematical model. The manuscript was written by KD and JM. AG reviewed the manuscript and gave scientific comments.

### Conflict of Interest

The authors declare that the research was conducted in the absence of any commercial or financial relationships that could be construed as a potential conflict of interest.
