# Supplementary material for: Induction of Phage-Specific Antibodies by Two Therapeutic Staphylococcal Bacteriophages Administered per os
Source: Front Immunol. 2019 Nov 14;10:2607. doi: 10.3389/fimmu.2019.02607 (PMC6871536; doi:10.3389/fimmu.2019.02607)
Supplement: Supplementary file 1 [file Data_Sheet_1.pdf]

Supplementary Materials

1 A3R and 676Z phage translocation from GI tract to circulation

It is unclear whether development of the phage-specific humoral response in the blood after oral administration requires translocation of active phage particles to the circulation. Nevertheless, in the context of oral application of phages as therapeutic agents in systemic infections, the ability of phages to penetrate through the gastrointestinal lining is particularly interesting. Therefore, we tested translocation of A3R and 676Z phage to blood. Detected translocation after 5 h and 27 h of treatment was poor and highly irregular (**Supplementary Figure 1**). Notably, even a phage dose 20 times higher than that used for oral immunization did not result in clearly demonstrated transfer of active phage to blood; it was highly irregular, including animals with almost 10^3 pfu/ml of blood and those without detectable phage. The detection limit in this experiment, due to the amount of blood that was possible to collect from one mouse, was 10^1 pfu/ml. Interestingly, a pH neutralizing agent applied prior to phage administration in drinking water did not result in improved phage penetration to blood (**Supplementary Figure 1**).

Irregular penetration of active phages to blood was also observed in the major experiment with prolonged administration of A3R and 676Z phage in drinking water (**Figure 1**). Phages were detectable in the blood, but their emergence was highly irregularly and in small titers, from zero up to 10^3 pfu/ml in some individuals (**Supplementary Figure 2**).

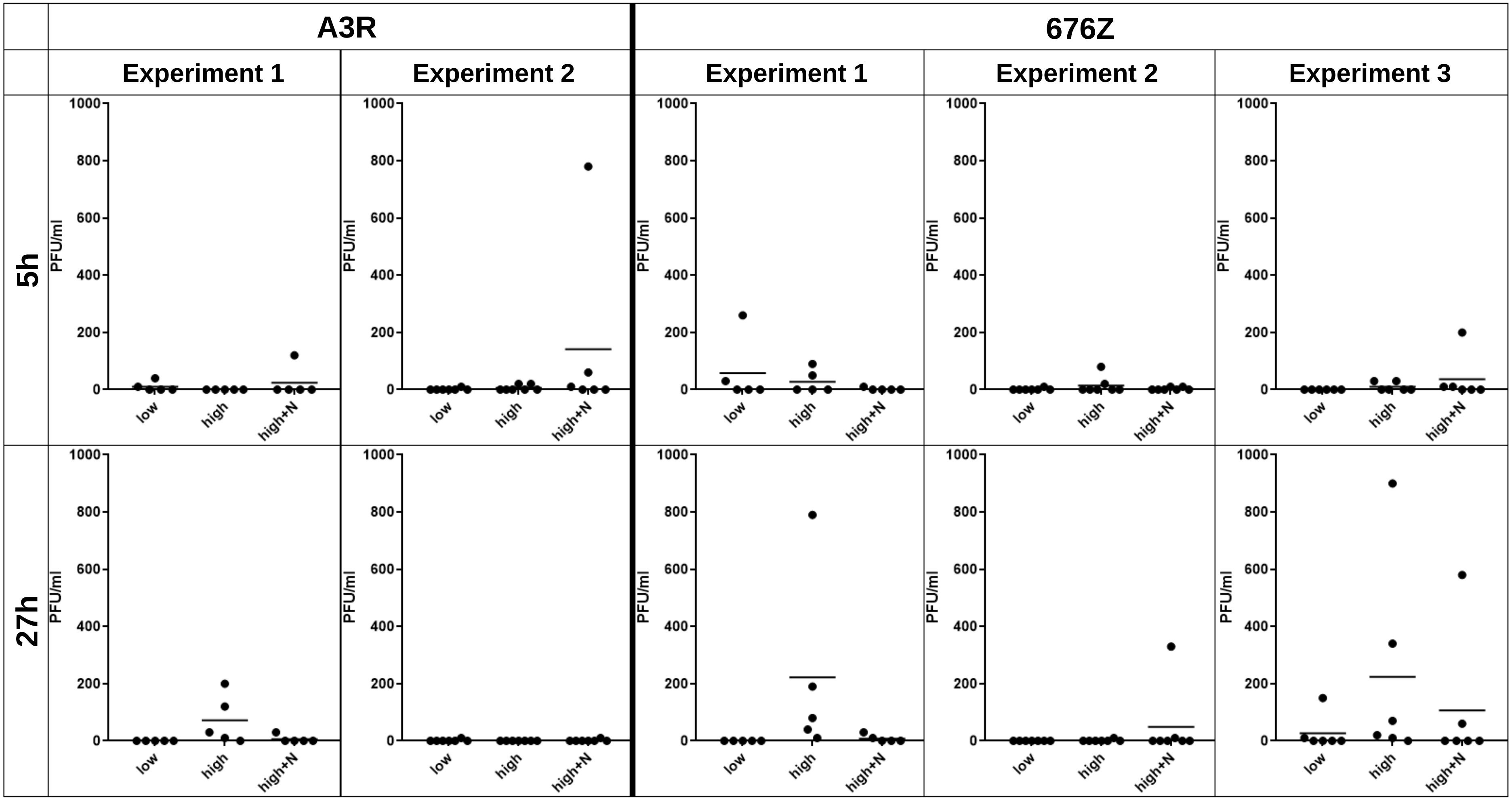

Supplementary Figure 1. A3R and 676Z phage titers detected in the blood of mice receiving phage preparations in drinking water for 5 h and for 27 h. Two different concentrations of phage particles were used: "low", i.e. 4×10^9 pfu/ml, and "high", i.e. 8×10^{10} pfu/ml; they were administered to mice (N=5-7) in drinking water. To determine whether neutralization of stomach acid affected phage translocation in these mice, mice receiving "high" phage titers ("high+N") were in addition given sodium bicarbonate in drinking water for 16 h before drinking water with phages. Due to highly irregular penetration all experiments (exp. 1-3) are presented.

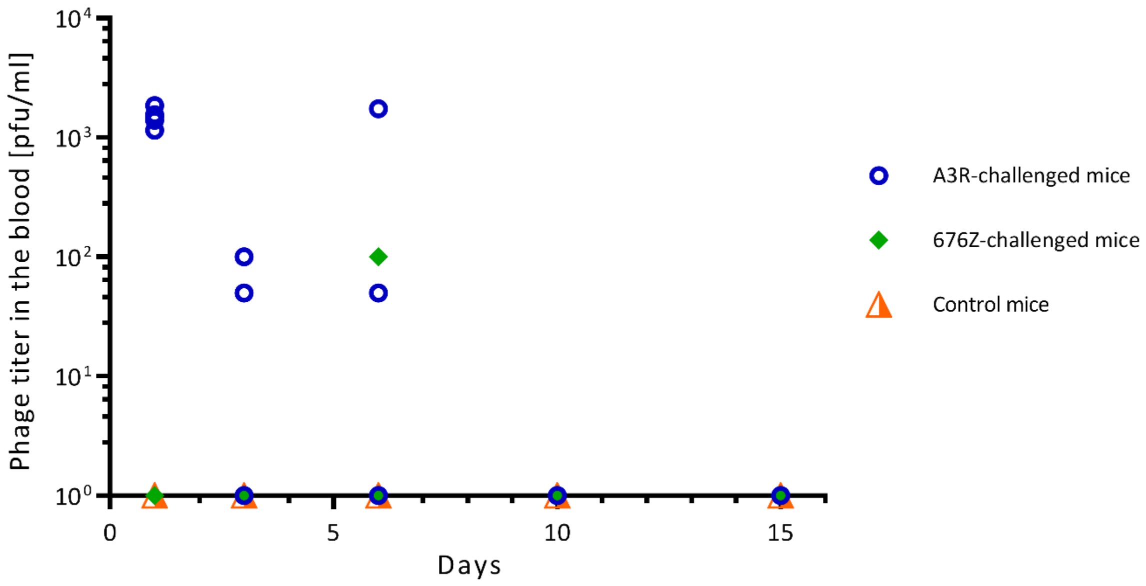

Supplementary Figure 2. A3R and 676Z phage titers detected in the blood of mice treated with phages A3R or 676Z. Mice (N = 7) were administered purified preparations of phages A3R or 676Z in drinking solution of the final concentration 4×10^9 pfu/ml. Phage-enriched drinking water was administered as a sole water source continuously for 100 days. For the following 120 days phage preparations were removed from the diet and then applied again for the final 44 days of the experiment. Only the first 15 days of the experiment are shown. No phages were detected in the blood for the rest of the experiment. Control mice were separated from phage-treated mice and received no phage in the diet. They were examined for presence of phages active against A3R and 676Z bacterial *S. aureus* host strains and no phage activity was detected during the whole experiment. Blood was collected from the tail vein; thus the same mice were sampled for the whole experiment. The experiment was repeated twice with concordant results. One representative experiment is presented in the figure.

2 Mathematical model

The raw dataset was composed of pairs $\{(t_i, x_i)\}_{i=0}^{N}$, where N stood for the total number of measurements, t_i was time of the i-th sample acquisition, and x_i stood for the amount of phage in blood. This was fitted to model pharmacokinetics of a *Myoviridae* phage in murine blood as previously identified by Kim et al. (Kim et al., 2008).

The first step of data preprocessing was normalization:

$$w_i \leftarrow \frac{x_i}{x_0} \tag{1}$$

and then logarithmic scaling was performed:

$$y_i = ln\, w_i \tag{2}$$

leading to the dataset $\{(t_i, y_i)\}_{i=0}^{N}$.

The whole process of mathematical modeling was performed using the logarithmic scale defined above. Visual inspection of the dataset revealed that the rate of decay of the variable y decreases with time. The function below accounted for this phenomenon:

$$y(t) = at^b, \tag{3}$$

where t is time, a and b are free-to-choose parameters. We determined the best values of the parameters using the least squares fit (Nocedal, 2006), see **Supplementary Figure 3**.

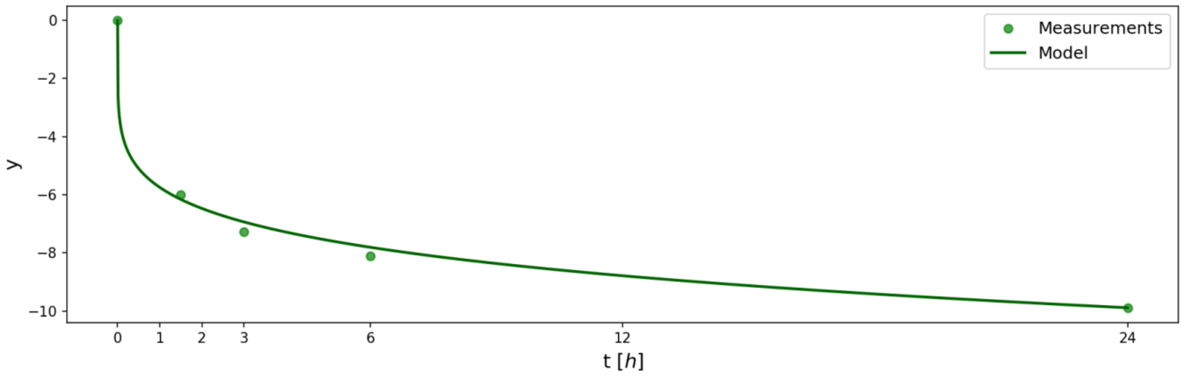

Supplementary Figure 3. Illustration of the model with parameters $a = -5.87, b = 0.17$.

For the purpose of prediction of the system state in response to phage injections, a description of dynamic properties of the process was needed. Calculation was done on the assumption that the underlying process was not distributed in space, thus the effect was immediate along the whole compartment represented by the variable x. Further assumption was that phage permeated continuously to the compartment, due to the constant exposure and activity of cells in healthy animals. Therefore, the differential equation was designed, that has a solution (3). This was an inverse problem to solving differential equation, where for a given differential equation the function satisfying it should be found; here, the differential equation behind it was designed. Among many differential equations having solution (3) we choose the one that allows for a good fit to the dataset. The equation had to accommodate possible phage injections. Thus, the resulting differential equation describing the whole process was:

$$x'(t) = a\frac{b}{t}t^{b}x(t) + u(t), \tag{4}$$

where $u(t)$ was the system input, which describes the way, the phage permeated the system. Note, that the state variable in differential equation (4) was x, but y as the model response was used. For the reason mentioned previously, simulations in this work assigned constant values for this function, resulting from transcytosis rate calculated according to Nguyen et al (Nguyen et al., 2017):

$$\Phi_{tr}^{day} = r_{tr}\, S_{li}\, \frac{\Phi_{li}}{V_{li}}\, 24h \tag{5}$$

$$\Phi_{tr,m}^{day} = mf \times \Phi_{tr}^{day} \tag{6}$$

where *mf* is the mucus factor equal to 4.4 as proposed by Nguyen et al. (Nguyen et al., 2017), and intestine surface (S_{li}) area was recalculated according to data given by Casteleyn et al. (Casteleyn et al., 2010). Initial phage concentration (Φ_{li}/V_{li} by Nguen et al. (Nguyen et al., 2017)) was calculated from experimental data on mean phage concentration in feces as observed within this work in an exemplary experiment (Experiment 1 in **Supplementary Figure 1**: A3R: 3.2×10^{7} pfu/ml, 676Z: 1.1×10^{8} pfu/ml). These allowed for calculation of

total phage amount expected to be transcytosed daily in investigated mice: **2.75x10^{6} pfu per 24h for A3R phage and 9.3x10^{6} pfu per 24h for 676 phage.**

The equation (4) allowed to simulate scenarios for given initial state of the process and different scenarios of phage dose available for transcytosis. Typical ones are illustrated in **Supplementary Figure 4** (of note, in logarithmic scale zero is represented by minus infinity and one corresponds to zero). Comparing two curves: the black one represents the process that started with a given amount of phage (normalized to 1) supplied continuously from outside (the input u is constant), the orange one represents the same process, but initiated with no phage (zero). In the latter case, phages enter the system only due to supply from outside the system.

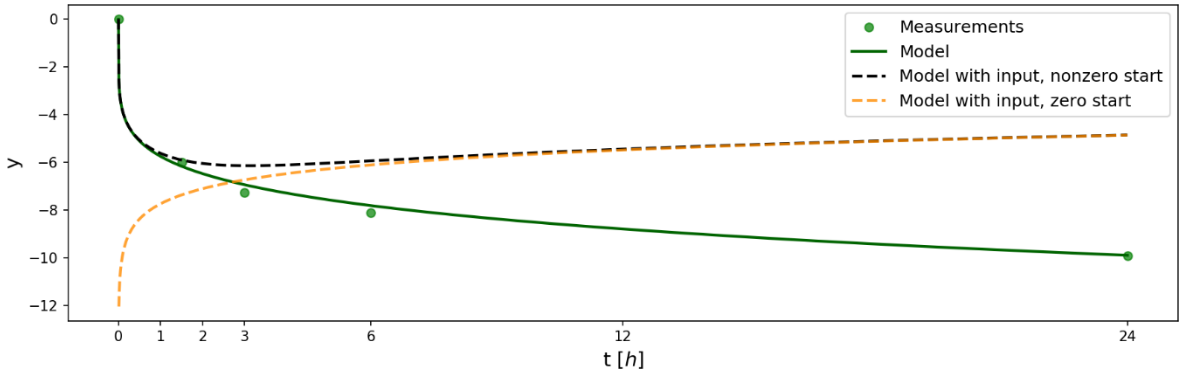

Supplementary Figure 4. Representative examples of simulations with- and without-administration and with zero- and nonzero- initial conditions (validation of the model).

The software was written in Python 3.7 with the *scipy* library to perform the least square fit of the function (2) and to simulate the process described by the differential equation (3).

Simulations allowed for calculation of expected phage concentration in blood, when assumed: (i) transcytosis rate according to Nguyen et al. (Nguyen et al., 2017), (ii) phage was distributed (diluted) in the whole body volume (not only in blood) which was an average 20 ml in these animals' groups, and (iii) phage concentration in the intestine was as identified in Experiment 1 (A3R: 3.2x10^{7} pfu/ml, 676Z: 1.1x10^{8} pfu/ml). As demonstrated by Denou et al. (Denou et al., 2009), effective transit of orally applied phage to lower sections of intestine can be completed within 4 hours, thus 27th hour of Experiment 1 is represented by 23rd hour in the simulation. Calculation of expected phage concentration

(over time) is presented in **Supplementary Figure 5** panel A3R and 676Z (orange line, normal). When compared to mean phage concentration in blood as observed in the Experiment 1 (orange line, dotted), expected phage concentration is approximately 1 order of magnitude higher than the observed one. Even maximum phage concentration observed in any individual mouse (grey line, dotted) was lower than the expected phage concentration. For discussion of this unexpected discrepancy please see Discussion section.

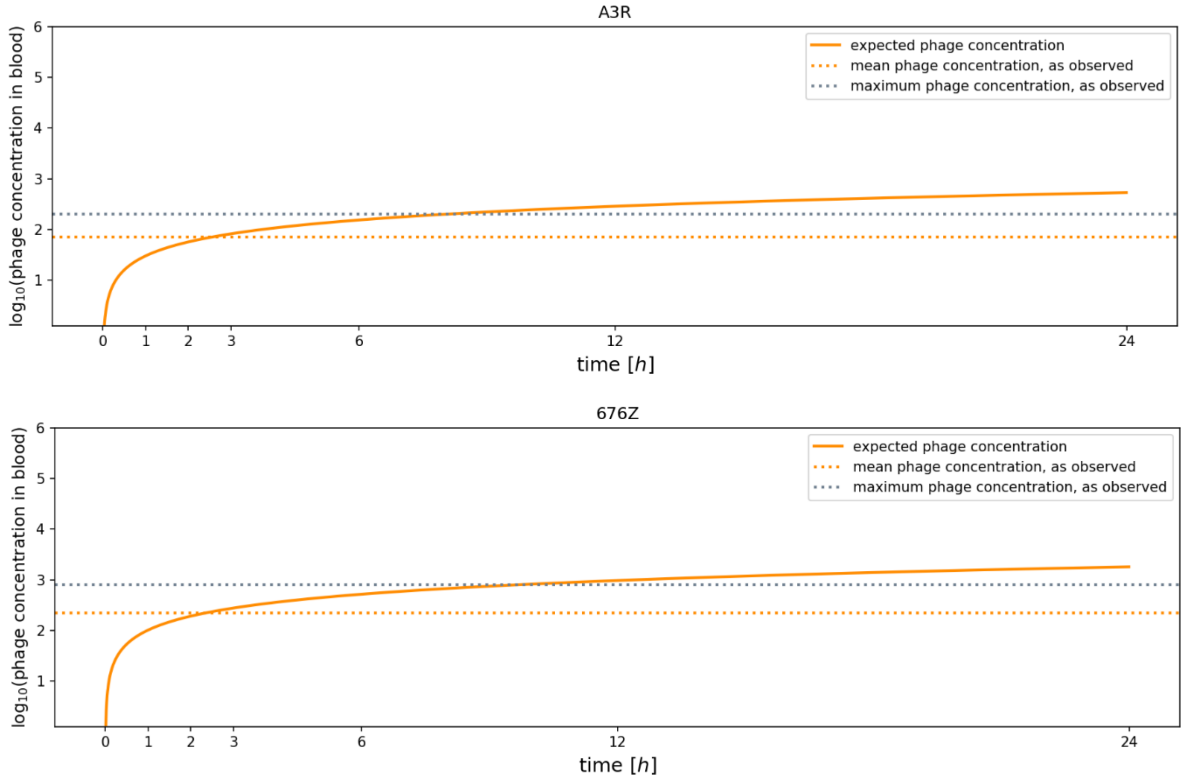

Supplementary Figure 5. Comparison of expected (simulated) and observed (experimental) phage concentrations in blood of mice fed with bacteriophages A3R and 676Z.

3 Microbiome assessment

Although much more specific than antibiotics, being antibacterial agents exogenous bacteriophages may still affect one's microbiome, especially when applied orally, as they come in contact with the highly complex commensal microflora of the GI tract. Studies have shown that shifts in the microbial composition (dysbiosis) in the gut may be linked to various diseases (6, 7). Therefore, the fecal bacterial microbiome composition was assessed by 16S rRNA targeted sequencing on day 1 and day 100, marking the end of the initial contact of experimental groups with bacteriophages A3R and 676Z. Analysis of the microbiome at the beginning of the experiment and at the end of the initial continuous phage treatment revealed no significant changes in alpha diversity with regard to either the Shannon-Wiener or the Simpson index (**Supplementary Table 1**). In the experimental groups a tendency to increase of Firmicutes class abundance was identified after 100 days **Supplementary Figure 6**). This seems to be similar to the tendency identified in humans, where the Firmicutes/Bacteroides ratio increased with increasing age (8). Since the experiments presented herein were very long, considering the murine life span, we suppose that the increase in Firmicutes probably represents normal changes in gut flora related to aging.

Supplementary Table 1. Average values of alpha diversity of the microbiome on day 1 and after 100 days of continuous phage treatment *per os* in drinking water.

	**Shannon-Wiener**		**Simpson**	
	**Day 1**	**Day 100**	**Day 1**	**Day 100**
**A3R-treated group**	2.82	2.12	0.735	0.6164
**676Z-treated group**	2.56	2.07	0.7332	0.6088
**Control group**	2.53	2.78	0.6984	0.7718

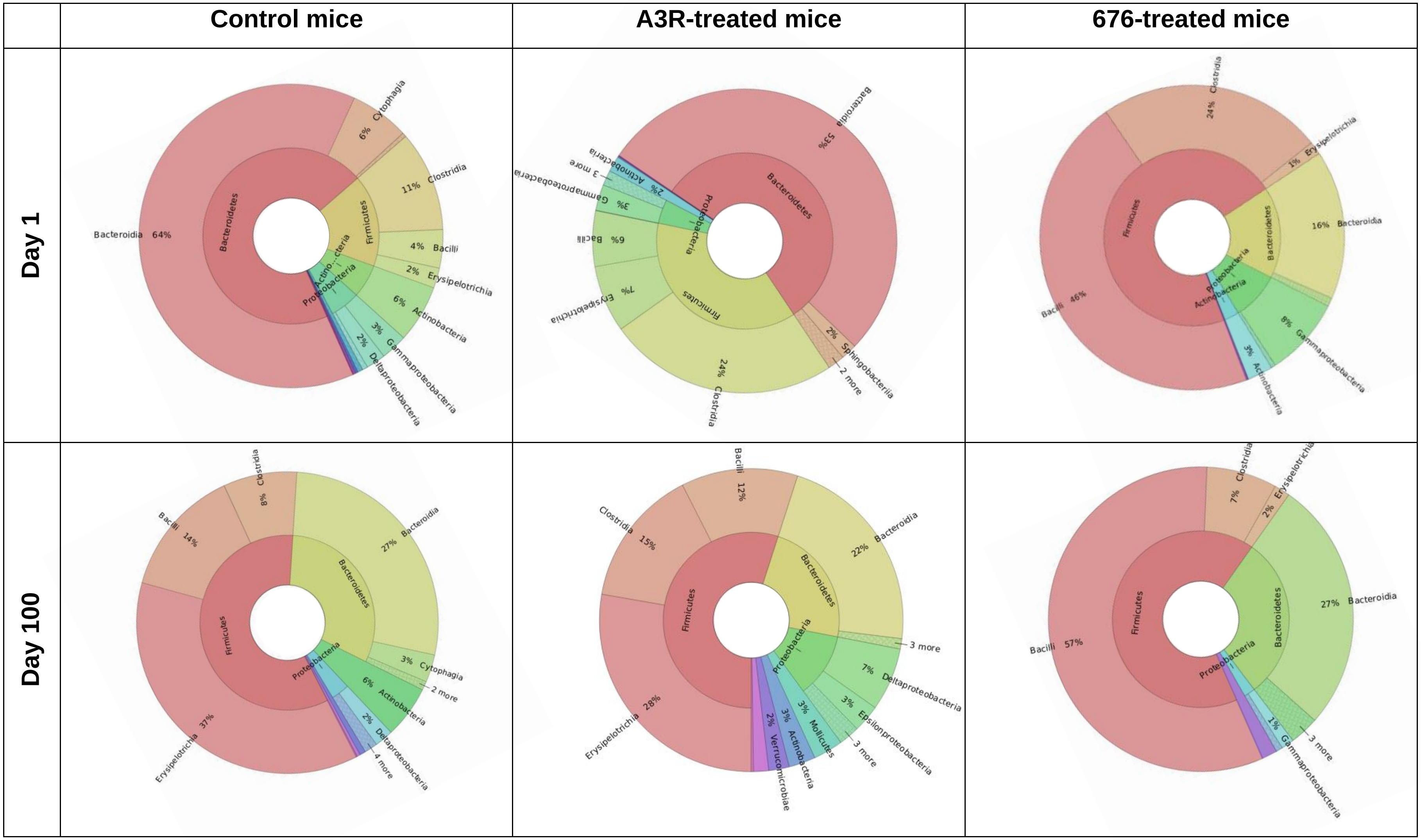

Supplementary Figure 6. Composition of bacterial microbiome component in mice on day 1 and after 100 days of continuous administration of A3R or 676Z phage preparations in drinking water.

4 Optimization of plasma dilution for blocking of phage activity by murine plasma samples

The plasma dilution factor used in the assay was determined experimentally. Following dilutions were tested: non-diluted samples, 1:10, 1:100, 1:1,000 and 1:2,000. For the final experiment 1:2,000 dilution was chosen as optimal (Fig. 4 in the main text of the manuscript), as for dilutions smaller than 1:1,000 after incubation with plasma samples from the experimental groups phage titers were reduced to undetectable levels, and for 1:1,000 dilution the differences between groups were unclear. Exemplary results are shown in **Supplementary Figure 7**.

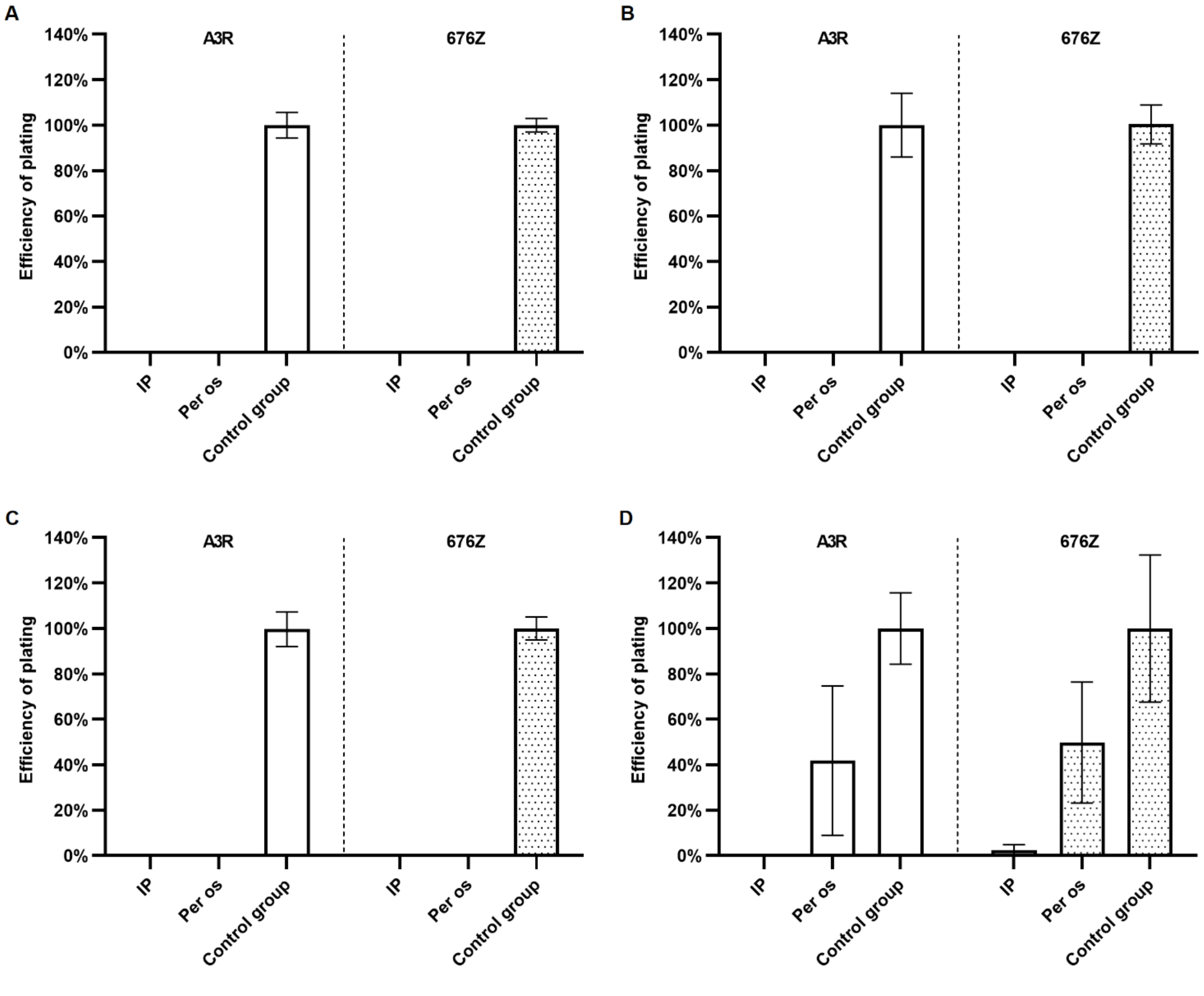

Supplementary Figure 7. Optimization of plasma dilution for comparison of blocking of phage activity by murine plasma samples: undiluted (A), 10-fold diluted (B), 100-fold diluted (C) and 1,000-fold diluted (D) plasma samples. Efficiency of plating of A3R and 676Z phages after incubation with plasma samples from mice treated with A3R or 676Z phage *per os* and intraperitoneally (IP). Mice (N = 5 or 6) were administered purified preparations of phages A3R or 676Z in drinking water as a sole

water source continuously for 100 days or they were injected with the phages intraperitoneally. For oral treatment a dose of 4×10^9 pfu/ml was used, thus making approx. 2×10^{10} pfu/mouse daily, $2x10^{12}$ in total. Intraperitoneal immunization was done with three successive injections of 1×10^{10} pfu/mouse on days 0, 20 and 50. EOP was tested for plasma samples collected on day 100. Exemplary results are presented.

5 References

Casteleyn, C., Rekecki, A., Van der Aa, A., Simoens, P., and Van den Broeck, W. (2010). Surface area assessment of the murine intestinal tract as a prerequisite for oral dose translation from mouse to man. *Lab Anim* 44(3)**,** 176-183. doi: 10.1258/la.2009.009112.

Denou, E., Bruttin, A., Barretto, C., Ngom-Bru, C., Brussow, H., and Zuber, S. (2009). T4 phages against Escherichia coli diarrhea: potential and problems. *Virology* 388(1)**,** 21-30. doi: 10.1016/j.virol.2009.03.009.

Kim, K.P., Cha, J.D., Jang, E.H., Klumpp, J., Hagens, S., Hardt, W.D., et al. (2008). PEGylation of bacteriophages increases blood circulation time and reduces T-helper type 1 immune response. *Microb Biotechnol* 1(3)**,** 247-257. doi: 10.1111/j.1751-7915.2008.00028.x.

Nguyen, S., Baker, K., Padman, B.S., Patwa, R., Dunstan, R.A., Weston, T.A., et al. (2017). Bacteriophage Transcytosis Provides a Mechanism To Cross Epithelial Cell Layers. *MBio* 8(6). doi: 10.1128/mBio.01874-17.

Nocedal, J., Wright, S. (2006). *Numerical optimization.* NY, USA: Springer Nature.
